# Bioresolution Production of (2*R*,3*S*)-Ethyl-3-phenylglycidate for Chemoenzymatic Synthesis of the Taxol C-13 Side Chain by *Galactomyces geotrichum* ZJUTZQ200, a New Epoxide-Hydrolase-Producing Strain 

**DOI:** 10.3390/molecules19068067

**Published:** 2014-06-16

**Authors:** Chun Wei, Jinlong Ling, Honglei Shen, Qing Zhu

**Affiliations:** College of Biological and Environmental Engineering, Zhejiang University of Technology, Hangzhou 310014, China; E-Mails: springweispring@163.com (C.W.); jor_ling@163.com (J.L.); shenhonglei-77@163.com (H.S.)

**Keywords:** ethyl 3-phenylglycidate, *Galactomyces geotrichum*, epoxide hydrolase, synthesis, bioresolution

## Abstract

A newly isolated *Galactomyces geotrichum* ZJUTZQ200 strain containing an epoxide hydrolase was used to resolve racemic ethyl 3-phenylglycidate (*rac*-EPG) for producing (2*R*,3*S*)-ethyl-3-phenylglycidate ((2*R*,3*S*)-EPG). *G. geotrichum* ZJUTZQ200 was verified to be able to afford high enantioselectivity in whole cell catalyzed synthesis of this chiral phenylglycidate synthon. After the optimization of the enzymatic production and bioresolution conditions, (2*R*,3*S*)-EPG was afforded with high enantioselectivity (*e.e._S_* > 99%, *E* > 49) after a 8 h reaction. The co-solvents, pH buffer solutions and substrate/cell ratio were found to have significant influences on the bioresolution properties of *G. geotrichum* ZJUTZQ200. Based on the bioresolution product (2*R*,3*S*)-EPG, taxol’s side chain ethyl (2*R*,3*S*)-3-benzoylamino-2-hydroxy-3-phenylpropionate was successfully synthesized by a chemoenzymatic route with high enantioselectivity (*e.e._S_* > 95%).

## 1. Introduction

Optically pure EPG, an important pharmaceutical intermediate, has a wide variety of applications. (2*R*,3*S*)-EPG is a key intermediate in the synthesis of taxol’s C-13 side chain ethyl (2*R*,3*S*)-3-benzoylamino-2-hydroxy-3-phenylpropionate [1]. It is also used in the synthesis of the drug reboxetine which is an inhibitor of the reuptake of norepinephrine [2]. In addition, (2*S*,3*R*)-EPG can be applied to the synthesis of the nootropic drug clausenamide [3]. Analogues of EPG also have a wide range of applications in drug synthesis. (2*R*,3*S*)-3-(4-methylphenyl)glycidic acid methyl ester is an important intermediate in the synthesis of the cardiovascular drug diltiazem [4]. Moreoever, (2*S*,3*R*)-benzylepoxy acid esters are important intermediates in the synthesis of a series of anti-cancer drugs that act as aminopeptidase N inhibitors, such as bestatin, phebestin and probestin, *etc.* [5].

The preparation of chiral phenylglycidate synthons is mainly achieved by chemical synthesis or enzymatic asymmetric hydrolysis [6]. Among the enzymatic methods, the use of lipase has been extensively reported. Mithilesh *et al.* obtained (2*R*,3*S*)-EPG with *e.e._S_* > 99% using Lecitase Ultra immobilized by gelatin as biocatalyst [7]. Zheng *et al.* prepared (2*R*,3*S*)-3-phenylglycidic acid methyl ester by hydrolyzing *rac*-3-phenylglycidic acid methyl ester using *Pseudomonas putida* cells as biocatalyst [8]. After the optimization of fermentation and catalytic conditions, the enantiomeric excess of the final product was greater than 99% with a yield of 48%. Manoucheh *et al.* obtained a series of chiral epoxy esters including chiral EPG through selective hydrolysis of the corresponding racemic compounds catalyzed by lipase from pig liver [9].

However, there are also several drawbacks in lipase-catalyzed asymmetric hydrolysis for producing chiral phenylglycidates, which may hinder its industrial application. For example, the unstable epoxy acid hydrolysis product may decarboxylate rapidly to the corresponding aldehyde [4], which affects the stability of the reaction system, and even leads to irreversible degenerative inactivation of lipase.

Another enzymatic option is epoxide hydrolase (EH) [10], which has none of the above drawbacks in the production of chiral phenylglycidates. In this field, EH-catalyzed hydrolysis of *rac*-EPG has been reported using the whole cells of *Pseudomonas* sp., yielding (2*R*,3*S*)-EPG with 95% *e.e._S_* and 26% yield in 12 h from 0.2% (w/v) of the racemate [11]. Since there are few reports on the preparation of chiral EPG by EH [11,12], the exploration of new microbial catalysts and the improvement of EH enantioselectivity are thus necessary.

**Scheme 1:**
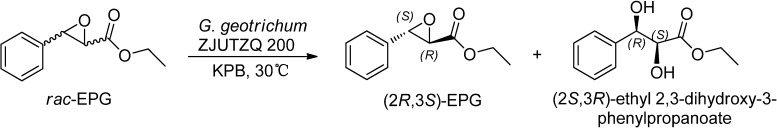
Enantioselective hydrolysis of *rac*-EPG by *G. geotrichum* ZJUTZQ200.

As a blockbuster anticancer drug, taxol represents one of the most clinically valuable natural products known to mankind in the recent past [1,13]. The dominant method for the preparation of taxol was the semisynthesis route by attaching a synthetic optically active C-13 side chain to natural baccatin III derived from renewable yew leaves. Chemoenzymatic synthesis protocols have been recognized as a very useful means to prepare optically active compounds [14,15]. Lipase-catalyzed asymmetric hydrolysis routes have been widely reported in the chemoenzymatic synthesis of taxol’s C-13 side chain [1]. In this work, we attempted to discover a new EH-producing microorganism that can be used to prepare chiral (2*R*,3*S*)-EPG as a synthon for taxol’s C-13 side chain. As a result, we obtained a newly isolated microorganism *Galactomyces geotrichum* ZJUTZQ200 to produce (2*R*,3*S*)-EPG with excellent enantioselectivity by whole-cell catalysis (Scheme 1). Furthermore, (2*R*,3*S*)-EPG prepared by EH was applied for the first time to the synthesis of the taxol C-13 side chain.

## 2. Results and Discussion

### 2.1. Screening and Identification of a Strain Producing Enantioselective EH

We have successfully isolated more than 200 strains from soil samples using 3-phenylglycidol, an analogue of *rac*-EPG, as the sole carbon source. The selective hydrolysis capabilities of *rac*-EPG were checked by HPLC. More than 30 strains showed EH activity. Among them, ten strains could preferentially hydrolyze (2*S*,3*R*)-EPG with high enantioselectivity leading to (2*R*,3*S*)-EPG configuration retention. One of the fungi strains, ZJUTZQ200, afforded the highest *e.e._S_* to produce (2*R*,3*S*)-EPG.

**Figure 1:**
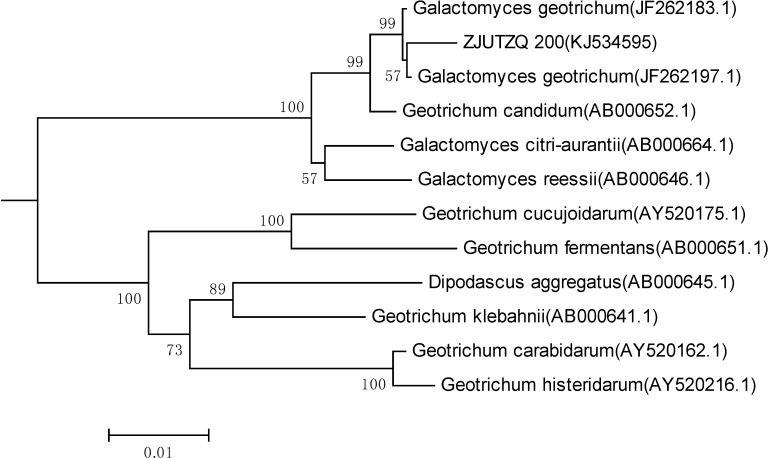
The phylogenetic tree based on 18S-ITS rDNA sequences, constructed by the neighbor-joining method, showing the relationship between the strain ZJUTZQ200 and the representatives of some related taxa.

The ITS sequence analysis of the strain ZJUTZQ200 was carried out [16]. The sequence data had been submitted to GenBank under the accession NO. KJ534595. A phylogenetic tree (Figure 1) was further constructed, and this strain was closely clustered with *Galactomyces geotrichum* (GenBank accession No. JF262197), having 99% sequence identity. Based on the results of phylogenetic analysis and phenotypic tests, the isolated strain ZJUTZQ200 was designated as *Galactomyces geotrichum* ZJUTZQ200 and deposited in the China Center for Type Culture Collection (CCTCC M 2013114). 

*G. geotrichum* was usually reported as a lipase producer. Its lipase also probably hydrolyzed EPG in the bioresolution reaction, but our results indicated little EPG was hydrolyzed by the lipase in *G. geotrichum* ZJUTZQ200 (data not shown). Hitherto, EH produced by *G. geotrichum* ZJUTZQ200 has never been reported.

### 2.2. Substrate Specificity of Chiral Resolution by G. geotrichum ZJUTZQ200

The structure of the substrate can significantly affect enzyme selectivity [17,18]. In order to identify substrate specificity, we investigated the substrate scope of EH in *G. geotrichum* ZJUTZQ200. Nine racemic epoxides, including five phenylglycidate compounds, were resolved by the whole cells of *G. geotrichum* ZJUTZQ200. As seen in Table 1, *G. geotrichum* ZJUTZQ200 exhibited a relatively high enantioselectivity (*e.e._S_* > 95%) towards phenylglycidate compounds **1**, **2**, **3** and **4**, suggesting that it had potential in producing chiral phenylglycidate synthons. The enantioselectivity of *G. geotrichum* ZJUTZQ200 towards compound **5** with a methoxy group substituent on the phenyl ring was significantly lower than that of other phenylglycidate compounds. The epoxides **6**, **7**, **8** and **9** were resolved with low enantioselectivity. The results indicated that chiral resolution by EH of *G. geotrichum* ZJUTZQ200 was specific to EPG and its derivatives.

**Table 1:** Enantioselective hydrolysis of *rac*-EPG and its analogues by *G. geotrichum* ZJUTZQ200.

No.	Substrate	Reaction Time (h) ^a^	Retention Configuration	*e.e._S_* (%) ^b^	*c* (%)	*E* Value ^c^
1	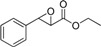	4	(2*R*,3*S*)	99.4	65	16
2	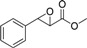	3	(2*R*,3*S*)	98.6	62.5	19
3	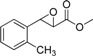	6	(2*R*,3*S*)	97.2	68	10
4	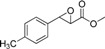	3	(2*R*,3*S*)	95.1	62.1	6
5	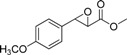	5	(2*R*,3*S*)	60.3	2.8	3
6	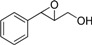	6	(2*R*,3*S*)	7.3	63.4	1
7	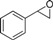	6	(2*R*,3*S*)	41.3	64.1	2
8	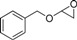	6	(2*S*,3*R*)	17.3	24.1	4
9	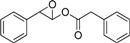	4	(2*S*,3*R*)	72.4	59	6

^a^ Racemic epoxide (5 mM, dissolved in DMSO) was hydrolyzed using wet mycelium of *G. geotrichum* ZJUTZQ200 (1 g) in 10 mL KPB (100 mM, pH 7.0) at 30 °C, 200 rpm for several hours; ^b^ The *e.e._S_* was calculated by the equation: *e.e._S_* = (*n_R,S_* − *n_S,R_*)/(*n_R,S_* + *n_S,R_*), where *n_R,S_* and *n_S,R_* are the concentrations of the (2*R*,3*S*)- and (2*S*,3*R*)-enantiomers, respectively; ^c^ The *E* value was enantiomeric selectivity which was calculated by the following equation: *E* = ln[(1 − *c*)(1 − *e.e._S_*)]/ln[(1 − *c*)(1 + *e.e._S_*)], *c* is the conversion rate of epoxide.

### 2.3. Optimization of Enzyme Production

In order to improve EH enzyme production, the culture conditions of *G. geotrichum* ZJUTZQ200, such as carbon and nitrogen sources, metal ions, the initial pH and culture time, *etc.* were optimized by single factor and orthogonal experiments. The optimal composition of enzyme production medium was as follows (per liter, pH 5.0): glycerin 10 g, soybean flour 15 g, NaNO_3_ 3 g, MgSO_4_ 0.5 g, KH_2_PO_4_ 1 g, KCl 0.5 g. The biomass with EH was improved to 34.3 g/L from the initial 8.2 g/L.

### 2.4. Effect of Co-solvents on the Resolution of Rac-EPG

**Figure 2:**
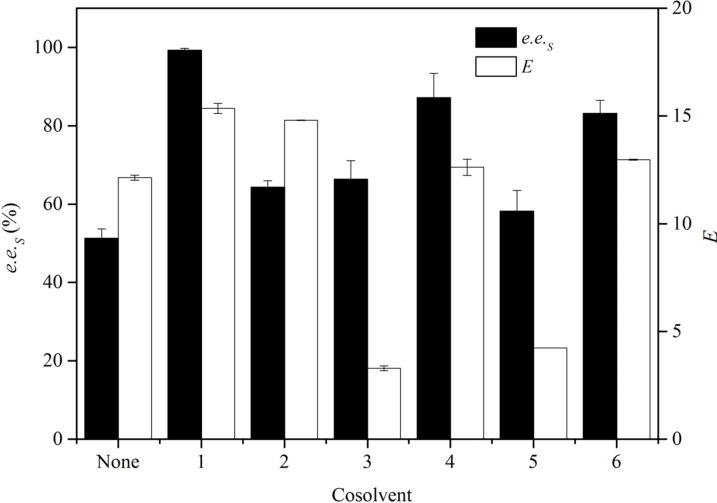
Effects of co-solvents on the resolution of *rac*-EPG by *G. geotrichum* ZJUTZQ200. Reaction conditions: 1.0 g wet mycelium, 0.01 mL *rac*-EPG, 10 mL KPB (100 mM, pH 7.0), 30 °C, 200 rpm, 6 h.

In enzymatic catalysis, a co-solvent can enhance the solubility of the substrate in water but also make it easier to get into the mycelium [19,20,21]. The effect of different organic solvents as co-solvents on the enantioselectivity was investigated at a concentration of 4‰ (v/v) (Figure 2). In comparison with the control without a supplementing co-solvent, the addition of different co-solvents all increased the *e.e._S_*. Higher *e.e._S_* and *E* values were obtained by the addition of DMSO, IPA and DMF. When acetone or ethanol was added, the conversion rate increased greatly so that *E* value was significantly decreased. In summary, DMSO as co-solvent afforded the best EH activity with the final *e.e._S_* of 99.3% and *E* of 15. Therefore, DMSO was chosen as the optimal co-solvent.

1: Dimethyl sulfoxide (DMSO), 2: tetrahydrofuran (THF), 3: acetone , 4: isopropanol (IPA), 5: ethanol, 6: N,N-dimethylformamide (DMF).

### 2.5. Effect of pH Buffer Solutions on Resolution of rac-EPG

**Figure 3:**
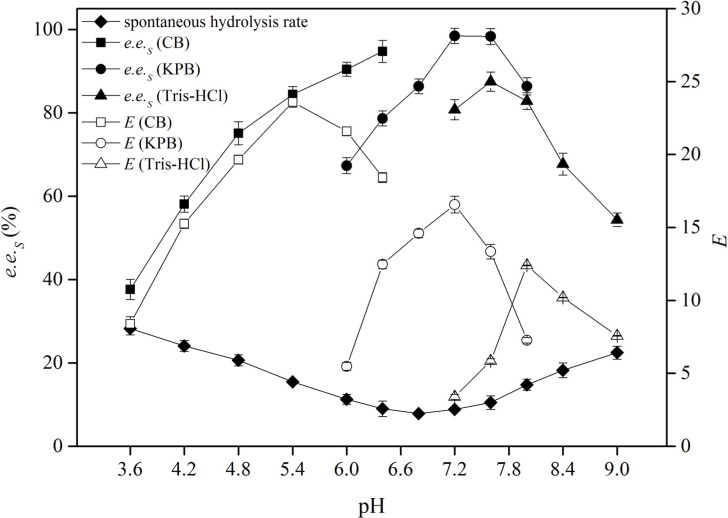
Effects of buffer types and buffer pH on the resolution of *rac*-EPG by *G. geotrichum* ZJUTZQ200. Reaction conditions: 1.0 g wet mycelium, 0.01 mL *rac*-EPG, 10 mL buffer, 30 °C, 200 rpm and 6 h.

The spatial conformation of the enzyme can be significantly affected by the type and pH value of the reaction buffer system [22], at the same time the dissociation situation of the substrate can also be influenced to some extent [23,24], thus affecting the binding of the enzyme and the substrate. To investigate the effects of pH and buffer type on the EH activity and enantioselectivity, the *e.e._S_* and *E* values were examined in different buffer systems (citrate buffer (CB), KPB and Tris-HCl) with a pH range from 3.6 to 9.0 at 30 °C (Figure 3). The spontaneous hydrolysis rate of *rac*-EPG in different buffer systems was also examined. The results indicated that the enantioselectivity of the epoxide hydrolase produced by *G. geotrichum* ZJUTZQ200 was very sensitive to pH, which was consistent with Hellström’s report [25]. In acidic and alkaline buffer systems, the spontaneous hydrolysis rate was high, while it was relatively low around neutral pH. In citrate buffer, the *e.e._S_* increased with pH and reached up to 94.7% at pH 6.4; meanwhile, the *E* value reached its maximum of 24 at pH 5.4. In KPB of pH 7.2, the *e.e._S_* and *E* all reached their maxima of 98.5% and 17; besides, the spontaneous hydrolysis rate was in the lowest level (<10%). In Tris-HCl buffer, the *e.e._S_* and *E values* were too low in comparison with the other two buffer systems, and the spontaneous hydrolysis rate was too high. As a result, KPB at pH 7.2 was chosen as the most favorable buffer solution.

### 2.6. Effect of Substrate/Cell Ratio on Resolution of rac-EPG

Due to the toxicity of the epoxide moiety to microbial cells and the transport resistance of biocatalysts [26], the concentration of substrate and the amount of biocatalyst have great effects on the enantioselectivity and reactivity of EH [27]. As shown in Table 2, when the substrate/cell ratio (%, w/w) was less than 7%, the *e.e._S_* of EPG could reach >99% after different bioconversion times. Under the condition of 10% mass ratio, the *e.e._S_* of EPG only reached 78.1%, even if the reaction time was prolonged. *E* value increased with the mass ratio and reached its maximum value of 49 at the mass ratio of 5%. The possible reason is that when the substrate concentration is low (EPG/mycelium < 3%), the catalytic center of EH is not saturated, resulting in low catalytic efficiency manifested as a low *E* value. High substrate concentration (EPG/mycelium > 7%) may inhibit the activity of the enzyme’s active center, meanwhile the hydrolysis product can change the pH value of the buffer leading to a decrease of the EH activity, thus resulting in low enantiomeric selectivity (*E* value). High *E* value and *e.e._S_* were all targets of biocatalysis optimization. *e.e._S_* above 99.5% was especially favorable in the medicinal synthon preparation. Thus, we selected the mass ratio of 5% as an optimal substrate/cell ratio.

**Table 2:** Effect of substrate/cell ratio on enantioselectivity of epoxide hydrolase in *G. geotrichum* ZJUTZQ200. Reaction conditions: 1.0 g wet mycelium, 10 mL KPB (100 mM, pH 7.2), 30 °C, 200 rpm.

Sub./Cell (%)	Reaction Time (h)	*e.e._S_* (%)	*c* (%)	*E* Vaule
1	4	99.4	75	11
2	6	99.5	71.7	12
3	8	99.8	72.5	28
5	8	99.5	69	49
7	8	99.8	75.1	24
10	13	78.1	74.7	3

### 2.7. Synthesis of Taxol’s C-13 Side Chain

**Scheme 2:**
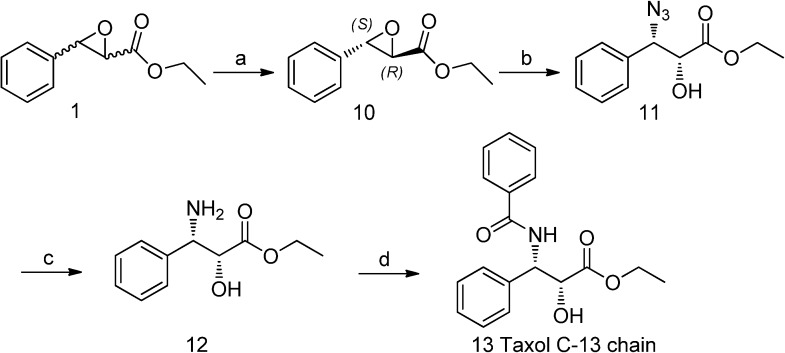
Preparation of taxol C-13 side chain from *rac*-EPG.

Taxol’s C-13 side chain is of notable importance in the drug’s powerful antileukemic and tumor-inhibiting activity [28]. A new synthetic strategy towards taxol’s C-13 side chain ethyl (2*R*,3*S*)-3-benzoylamino-2-hydroxy-3-phenylpropionate (**13**) is shown in Scheme 2. Firstly, based on the optimal resolution process with *G. geotrichum* ZJUTZQ200, optically pure (2*R*,3*S*)-EPG (*e.e._S_* > 99%) was obtained in a yield of 37.1%. Secondly, (2*R*,3*S*)-EPG was reacted with sodium azide in methanol to afford **11** in a yield of 95%. Subsequently, the reduction reaction with triphenylphosphine in THF to afford **12**, followed by acylation with benzoyl chloride to obtain the desired ethyl (2*R*,3*S*)-3-benzoylamino-2-hydroxy-3-phenylpropionate (**13**, *e.e._S_* > 95%) in a total yield of 33.8%. No racemization was observed during the whole formation of taxol’s C-13 side chain.

*Reaction conditions*: (a) *G. geotrichum* ZJUTZQ200, KPB; (b) NaN_3_, NH_4_Cl, methanol, 65 °C; (c) triphenyl phosphine, THF, rt; (d) benzoyl chloride, dichloromethane, rt.

## 3. Experimental Section

### 3.1. Materials

*rac*-EPG **1** and microbial media components were purchased from J&K Scientific Ltd. (Shanghai, China). (2*R*,3*S*)-EPG was purchased from UHN Shanghai R&D Co., Ltd. (Shanghai, China). Other chemicals used were of analytical reagent grade and purchased from various commercial sources.

### 3.2. Preparation of Derivatives of EPG

Phenylglycidates **2**, **3**, **4** and **5** were synthesized by Darzens reaction [29]. Briefly, methyl chloroacetate (10 mmol) was dissolved in a solution of sodium methoxide (10 mmol) in dry methanol (10 mL) under ice bath cooling and stirred for 5 min, followed by the addition of corresponding aldehyde (5 mmol). The mixture was kept at 0 °C with stirring until completion and then the methanol was removed under reduced pressure. Dichloromethane (40 mL) was added and then washed with saturated sodium chloride solution (50 mL × 3), dried over anhydrous sodium sulfate and concentrated under vacuum and finally purified by thin layer chromatography (TLC) to give the desired derivatives of EPG. TLC was performed on F254 silica gel plates with the eluent petroleum ether/ethyl acetate (10:1, v/v). All the synthesized substrates were confirmed by LC-MS and ^1^H-NMR. NMR spectra were recorded on a Digital Avance 400 MHz spectrometer (Bruker) using tetramethylsilane as an internal standard.

*Methyl 3-phenylglycidate* (**2**): pale yellow liquid, yield 37%, ^1^H-NMR (CDCl_3_) δ 7.25 (s, 5H), 4.01 (s, 1H), 3.70 (d, *J* = 9.1 Hz, 3H), 3.42−3.39 (m, 1H). MS (ESI) *m/z* 178.0 (M)^+^.

*Methyl 3-(2-methylphenyl) glycidate* (**3**): pale yellow liquid, yield 42%, ^1^H-NMR (CDCl_3_) δ 7.05 (s, 2H), 6.72 (dd, *J* = 6.8, 1.9 Hz, 2H), 3.94 (s, 1H), 3.68–3.62 (m, 6H), 3.41 (s, 1H). MS (ESI) *m/z* 192.1 (M)^+^.

*Methyl 3-(4-methylphenyl) glycidate* (**4**): pale yellow liquid, yield 36%, ^1^H-NMR (CDCl_3_) δ 7.06 (s, 4H), 3.96 (d, *J* = 1.7 Hz, 1H), 3.96 (d, *J* = 1.7 Hz, 3H), 3.42 (s, 1H), 2.25 (s, 3H). MS (ESI) *m/z* 192.1 (M)^+^.

*Methyl 3-(4-methoxyphenyl) glycidate* (**5**): pale yellow liquid, yield 41%, ^1^H-NMR (CDCl_3_) δ 7.15–7.05 (m, 4H), 4.14 (s, 1H), 3.75 (s, 3H), 3.33 (s, 1H), 2.31 (s, 3H). MS (ESI) *m/z* 208.2 (M)^+^.

### 3.3. Screening of Strains and Enantioselective Hydrolysis to Rac-EPG

Soil samples were used for the screening of strains capable of utilizing 3-phenylglycidol as a sole carbon source. Isolated strains were transferred to 50 mL basal enzyme production medium and cultivated at 200 rpm, 30 °C for 48 h for bacteria, or 72 h for molds. After the cells were harvested, 1 g wet cells were washed and suspended in 10 mL 0.1 M, pH 7.0 potassium phosphate buffer (KPB). Cell suspensions were mixed with 50 μL substrate solution (EPG:DMSO = 2:8,V/V) and kept at 200 rpm, 30 °C for 6 h reaction, then the optical purity of the remaining substrate was determined by chiral HPLC. The screening medium contained (per liter, pH 6.5) 10 g soybean flour, 2 g NaNO_3_, 0.5 g KCl, 0.5 g MgSO_4_·7H_2_O, 1 g K_2_HPO_4_·3H_2_O, and 0.1% (v/v) 3-phenylglycidol. Basal enzyme production medium contained (per liter, pH 6.5) 10 g soybean flour, 2 g NaNO_3_, 0.5 g KCl, 0.5 g MgSO_4_·7H_2_O, 1 g K_2_HPO_4_·3H_2_O, and 30 g sucrose.

### 3.4. Identification of the Strain ZJUTZQ200

The isolated strain was preliminarily identified by morphological and microscopic observation. Furthermore, its 18S-internal transcribed spacer (ITS) regions of the rDNA were obtained through gene sequencing. The 18S-ITS region sequence was deposited in the GenBank database. Related sequences were obtained from GenBank database (National Center for Biotechnology Information) using the BLAST system. The 18S-ITS regions determined were aligned with the reference sequences obtained from GenBank databases using ClustalW ver.1.81 [30]. MEGA ver.5.1 was applied for the calculation of evolutionary distance and finally a phylogenetic tree was constructed using the neighbor-joining method [31,32].

### 3.5. Analytical Methods

The commercially available (2*R*,3*S*)-EPG was employed as a standard to identify the absolute configuration. The absolute configurations of the other products were assigned by comparing the measured specific rotations and retention times on chiral HPLC columns with the reported ones. EPG and its analogues were detected by HPLC (Waters 1525) equipped with a Daicel Chiralpack AS-H column (0.46 cm × 25 cm, 5 μm). The mobile phase was a mixture of *n*-hexane and isopropanol (8:2, v/v) at a flow rate of 0.8 mL/min. The value of *e.e._S_* was expressed as enantiomeric excess of the remaining epoxide, which was calculated using the following equation: *e.e._S_* = (*n_R,S_* − *n_S,R_*)/(*n_R,S_* + *n_S,R_*) (*n_R,S_* and *n_S,R_* were the concentrations of the (2*R*,3*S*)- and (2*S*,3*R*)-enantiomer, respectively). *E* value was enantiomeric ratio of epoxides, which was calculated using the following equation: *E* = ln[(1 − *c*)(1 − *e.e._S_*)]/ln[(1 − *c*)(1 + *e.e._S_*)] (*c*, conversion rate) [33].

### 3.6. Synthesis of Taxol’s C-13 Side Chain

*(2R,3S)-EPG* (**10**): *rac*-EPG (1 mL) in DMSO (4 mL, 2:8, v/v) was dissolved in KPB (195 mL, 100 mM, pH 7.2) using wet mycelium of *G. geotrichum* ZJUTZQ200 (20 g) as biocatalyst at 30 °C (oil bathing) by stirring with a magnetic stir bar at 200 rpm. After 8 h biotransformation, the reaction solution was centrifuged to remove the mycelium and then extracted by *n*-butanol (60 mL × 3). The combined organic phase was concentrated under reduced pressure, followed by TLC purification with the eluent petroleum ether/ethyl acetate (10:1, v/v). 0.36 g (2*R*,3*S*)-EPG (*e.e._S_* > 99%) was obtained. 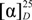 = −148.6 (*c* 2, CHCl_3_) {lit. [34] 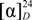 = −158.8 (*c* 1.06, CHCl_3_)}.

*(2R,3S)-3-azido-2-hydroxy-benzenepropanoic acid ethyl ester* (**11**): (2*R*,3*S*)-EPG (0.25 g, 1.3 mmol) was dissolved in methanol (10 mL) in round-bottom flask followed by addition of sodium azide (0.25 g, 3 eq.) and ammonium chloride (0.19 g, 3 eq.), then the mixture was stirred at 65 °C for 6 h. After completion of the reaction, the solvent was removed under reduced pressure and redissolved in ethyl acetate (30 mL). The organic phase was washed with saturated sodium chloride (2 × 30 mL) and dried over anhydrous sodium sulfate and then concentrated under reduced pressure to give 0.3 g (95% yield) of **11** as a pale yellow oil. ^1^H-NMR (CDCl_3_): δ 7.30 (s, 5H), 4.83 (s, 1H), 4.46 (s, 1H), 4.13 (s, 2H), 2.70 (s, 1H), 1.14 (s, 3H). MS (ESI) *m/z* 235.1 (M)^+^. 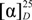 = +141.1 (*c* 1.8, CHCl_3_) {lit. [35] 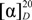 = +133.5 (*c* 2, CH_2_Cl_2_)}.

*(2R,3S)-3-amino-2-hydroxy-3-phenylpropionic acid ethyl ester* (**12**): Compound **11** (0.3 g, 1.3 mmol) and triphenylphosphine (1 g, 3 eq.) were dissolved in tetrahydrofuran (30 mL) and then stirred at room temperature for 6 h. The solvent was removed under reduced pressure and then purified by TLC (eluent petroleum ether/ethyl acetate 2:1, v/v) to obtain **12** as pale yellow oil. ^1^H-NMR (CDCl_3_): δ 7.25–7.18 (m, 5H), 4.43 (s, 1H), 4.34 (s, 1H), 4.05–3.95 (m, 2H), 2.58 (s, 3H), 1.14 (s, 3H). MS (ESI) *m/z* 209.0 (M)^+^. 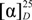 = −4 (*c* 1, CHCl_3_) {lit. [36] 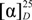 = −2.9 (*c* 0.26, CHCl_3_)}.

*Ethyl (2R,3S)-3-benzoylamino-2-hydroxy-3-phenylpropionate* (**13**): Triethylamine (0.72 g, 1.5 eq.) was dissolved in a solution of **12** (0.1 g, 0.5 mmol) and benzoyl chloride (0.77 g, 1.5 eq.) in dichloromethane (20 mL) and then stirred at room temperature for 4 h. The solvent was removed under reduced pressure and then purified by TLC (eluent petroleum ether/ethyl acetate 2:1, v/v) to give 0.102 g of **13** (*e.e._S_* > 95%) as white solid with a total yield of 33.8%. ^1^H-NMR (CDCl_3_): δ 7.68 (d, *J* = 8.4 Hz, 1H), 7.37–7.14 (m, 10H), 5.51 (dd, *J* = 8.5, 3.5 Hz, 1H), 4.55 (d, *J* = 3.5 Hz, 1H), 4.07–3.95 (m, 2H), 3.41 (s, 1H), 1.12 (t, *J* = 7.1 Hz, 3H). MS (ESI) *m/z* 336.1 (M)^+^. 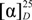 = −11.4 (*c* 1, CHCl_3_) {lit. [37] 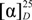 = –12 (*c* 2, CHCl_3_)}.

## 4. Conclusions

Bioresolution of *rac*-EPG is one of the important potential synthetic routes for industrial production of enantiopure (2*R*,3*S*)-EPG. In this work, we obtained a new strain capable of resolving *rac*-EPG to (2*R*,3*S*)-EPG with high enantioselectivity and identified it as *G. geotrichum* ZJUTZQ200. The use of this strain in enantioselective synthesis of (2*R*,3*S*)-EPG has not been previously reported. The substrate scope investigation results indicated *G. geotrichum* ZJUTZQ200 could afford high enantioselectivity in the synthesis of the chiral phenylglycidate synthon, suggesting that it had good potential in industrial applications. After the optimization of enzyme production and the whole cell catalysis process, *G. geotrichum* ZJUTZQ200 could resolve *rac*-EPG to (2*R*,3*S*)-EPG with *e.e._S_* of 99.5% and *E* value of 49, both of which were higher than those reported in the literature for (2*R*,3*S*)-EPG preparation by enantioselective synthesis using whole cells containing EH [11,12]. Co-solvents, pH buffer solutions and substrate/cell ratio significantly affected the enantioselectivity of *G. geotrichum* ZJUTZQ200. Especially, the *E* value was improved to 49 from 17 at a substrate/cell ratio of 5%.

As a result, based on the optimized process of (2*R*,3*S*)-EPG synthesis by *G. geotrichum* ZJUTZQ200, we established a new chemoenzymatic synthesis route to produce taxol’s C-13 side chain, affording *e.e._S_* of 95% and a total yield of 33.8%. In comparison with the chemoenzymatic synthesis route based on lipase-catalyzed asymmetric hydrolysis, our scheme is a promising alternative for the industrial preparation of taxol’s C-13 side chain due to EH’s advantages.
